# Synthesis and Characterization of a New Cryogel Matrix for Covalent Immobilization of Catalase

**DOI:** 10.3390/gels8080501

**Published:** 2022-08-12

**Authors:** Canan Altunbaş, Ahmet Aslan, Kevser Kuşat, Mehtap Sahiner, Sinan Akgöl, Nurettin Sahiner

**Affiliations:** 1 Department of Biochemistry, Faculty of Science, Ege University, Izmir 35100, Turkey; 2 Department of Leather Engineering, Faculty of Engineering, Ege University, Izmir 35100, Turkey; 3 Department of Chemistry, Faculty of Science, Dokuz Eylul University, Izmir 35390, Turkey; 4 Bioengineering Department, Faculty of Engineering, Canakkale Onsekiz Mart University, Terzioglu Campus, Canakkale 17100, Turkey; 5 Department of Chemistry, Faculty of Sciences & Arts, Nanoscience and Technology Research and Application Center (NANORAC), Canakkale Onsekiz Mart University, Terzioglu Campus, Canakkale 17100, Turkey; 6 Materials Science and Engineering Program, Department of Chemical & Biomedical Engineering, University of South Florida, Tampa, FL 33620, USA; 7 Department of Ophthalmology, University of South Florida, Tampa, FL 33620, USA

**Keywords:** enzyme immobilization, cryogel, catalase, superporous polymer network

## Abstract

The advantages of cryogels for enzyme immobilization applications include their mechanical and chemical robustness, ease of production, superior porosity, and low cost. Currently, many researchers are exploring porous material-based systems for enzyme immobilization that are more efficient and economically viable. Here, poly(2-Hydroxyethyl methacrylate-co-allyl glycidyl ether) (p(HEMA-co-AGE)) cryogel matrices were synthesized via the free radical cryopolymerization method to be employed as the support material. For the immobilization of the catalase enzyme onto the p(HEMA-co-AGE) cryogel matrix (catalase@p(HEMA-co-AGE), the best possible reaction conditions were determined by altering parameters such as pH, catalase initial concentration, and flow rate. The maximum catalase immobilization amount onto the p(HEMA-co-AGE) cryogel was found to be 48 mg/g cryogel. To determine the advantages of the cryogel matrix, e.g., the stability and reusability of the cryogel matrix, the adsorption–desorption cycles for the catalase enzyme were repeated five times using the same cryogel matrix. At the end of the reusability tests, it was found that the cryogel was very stable and maintained its adsorption capacity with the recovery ratio of 93.8 ± 1.2%. Therefore, the p(HEMA-co-AGE) cryogel matrix affords repeated useability, e.g., up to five times, without decreasing its catalase binding capacities significantly and has promising potential for many industrial applications. Cryogels offer clear distinctive advantages over common materials, e.g., micro/nano particles, hydrogels, films, and composites for these applications. At present, many researchers are working on the design of more effective and economically feasible, porous material-based systems for enzyme immobilization

## 1. Introduction

Many support materials used in enzyme immobilization have been investigated in the literature to date [1,2,3]. Micron-size particles, membranes, and nanoparticles are the most common materials employed in immobilization studies [4,5,6]. However, new materials with versatile properties and multitask abilities are always sought. One of the new classes of materials for enzyme immobilization is cryogels, which have significant potential in biotechnology [7]. Cryogels are an excellent alternative for the immobilization of not just enzymes, but also other biomolecules such as polysaccharides, proteins, hormones, nucleic acids, and so on due to their intriguing and modifiable features such as interconnected supermacropores, short diffusion paths and high mass transfer ability, abundant functionality and biocompatibility, etc. [8].

Conventional packed-bed columns have some inherent limitations, such as a slow diffusional mass transfer and large void volume between the column support material [9]. Approaches using non-porous polymeric beads [10] are one of the alternatives that have been developed to overcome these drawbacks. Another alternative is perfusion chromatography applications, which are recommended to be used as stationary phases. None of these alternatives developed could completely eliminate these problems [11]. The innate characteristics of supermacroporous polymeric structures include a high water content, non-toxicity, user-friendliness, adaptability, and efficiency. Moreover, the reusability and effortless storage-ability (no specific need) render added advantages. Therefore, cryogels can be used in many different biomedical applications including drug delivery, biomolecule immobilization, separation, and tissue engineering due to their better mechanical and chemical robustness, easy production, superior porosity, low cost, and so on. Cryogels offer clear distinctive advantages over common materials, e.g., micro/nano-particles, hydrogels, films, and composites for these applications. At present, many researchers are working on the design of more effective, economically feasible, and porous material-based systems for enzyme immobilization [12,13,14].

Catalase is a well-known enzyme that catalyzes the breakdown reaction of hydrogen peroxide or, in other words, a protein molecule which breaks down 5 × 10^6^ hydrogen peroxide molecules per minute [15]. There are also many industrial applications where catalase is used, for example, in the removal of hydrogen peroxide from dairy processing or bleaching in the textile industry for dyeing [16,17,18]. In addition, the catalase enzyme can be used in many fields as part of hydrogen peroxide and glucose–biosensor systems [19,20,21].

In this study, we have developed a new cryogel material, p(HEMA-co-AGE), for catalase immobilization. The synthesis and characterization studies of the p(HEMA-co-AGE) cryogel were carried out by employing various instrumental techniques such as FTIR-ATR, SEM, BET, XPS, as well as swelling tests. The covalent bonding of the catalase enzyme and cryogel, as the immobilization of the catalase enzyme onto the p(HEMA-co-AGE) cryogels resulted in catalase@p(HEMA-co-AGE), was also investigated. Firstly, the optimum binding conditions to obtain catalase@p(HEMA-co-AGE) in a continuous system were investigated. The activity of the free enzyme and catalase@p(HEMA-co-AGE) was compared to assess the advantages of the immobilization method. The effects of the initial concentration of catalase and pH on the activity of the catalase@p(HEMA-co-AGE) cryogel system and free catalase were compared. This study indicates that the enzyme catalase can easily be immobilized on p(HEMA-co-AGE) cryogels, which are hydrophobic due to their AGE units with free epoxide groups available for immobilization. This cryogel’s structure, developed for the immobilization of catalase, showed enhanced results at different pHs and temperatures than free catalase. The activity measurements for free and immobilized catalase were carried out using hydrogen peroxide as a substrate and a comparison of the Km and Vmax values was performed using Lineweaver–Burk plots. Moreover, the reusability of the catalase@p(HEMA-co-AGE) system in the degradation of H_2_O_2_ was also studied.

## 2. Results and Discussion

### 2.1. Characterization of p(HEMA-co-AGE) Cryogels

**Figure 1:**
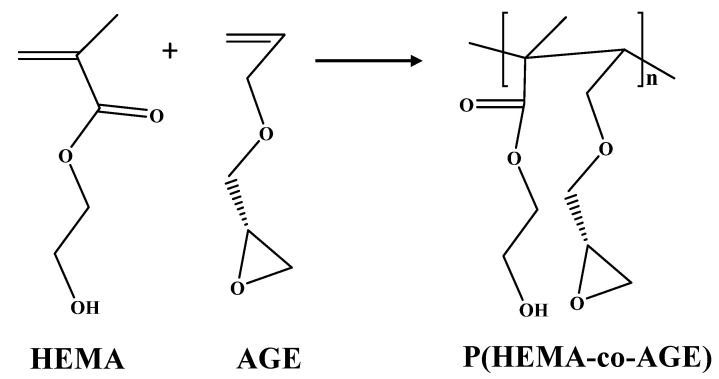
Possible reaction mechanism of HEMA and AGE monomers resulting in the formation of p(HEMA-co-AGE) cryogels.

Cryogels offer promising alternatives to traditional protein-binding materials due to their high blood compatibility and high water retention rate. Additionally, the cryogels are porous, have no toxicity, and show resistance to degradation and pressure drops, allowing them to be used frequently in the medical field without any diffusion problems when working with biological macromolecules. The applications of cryogel matrices in the immobilization of biomolecules, capture of target molecules, controlled drug release, cell separation, scaffolding, and in bioreactors afford new avenues for their biotechnological use. PHEMA is a well-known and FDA-approved acrylate polymer with many applications including contact lenses, drug delivery, separation, and implants [22,23,24]. Allyl glycidyl ether has a pendant epoxy group with allylic ether. By using these two monomers, the hydroxyl and epoxide functional group-containing structure can be prepared as a functional (co)polymeric material to be further use by the utilization of these functionalities [25]. Figure 1 shows the schematic presentation of the reaction mechanism of the p(HEMA-co-AGE) cryogel. As the epoxide groups on the p(HEMA-co-AGE) cryogel can be utilized for the conjugation of biological molecules such as proteins and enzymes, here, we have chosen HEMA and AGE monomers to prepare porous cryogels to be conjugated with enzymes such as catalase.

**Figure 2:**
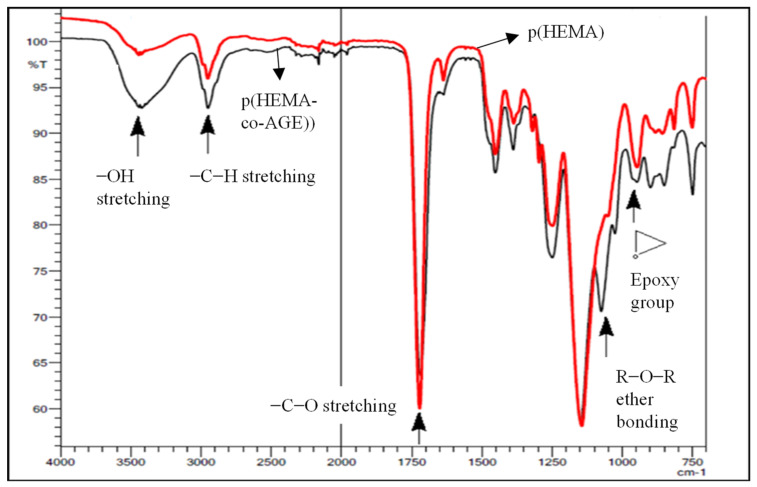
FTIR−ATR spectra of p(HEMA) and p(HEMA-co-AGE) cryogels.

Figure 2 shows the FTIR-ATR spectra of the p(HEMA) and p(HEMA-co-AGE) cryogels. The vibrations belonging to the –OH group on the structure p(HEMA) were seen at 3500 cm^−1^, the stretching vibration of the C-H group in the p(HEMA) and p(HEMA-co-AGE) structures could also be clearly seen at 3050 cm^−1^, the stretching vibrations were at 1750 cm^−1^ for C=O (ester carbonyl) from HEMA, and the stretching at 1050 cm^−1^ belonged to R-O-R (ether bond) coming from AGE. The existence of the stretching vibrations belonging to the epoxy group at 915 cm^−1^ also collaborated with the study conducted by Nechaeva et al. [26].

**Figure 3:**
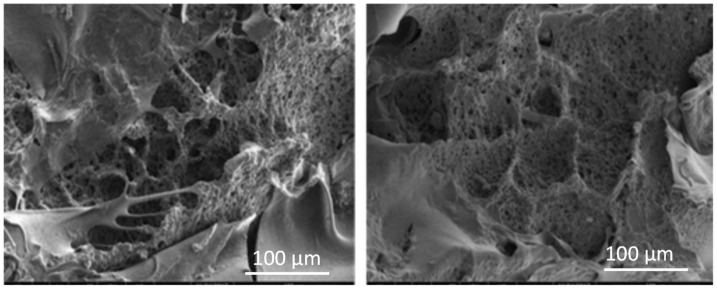
SEM micrographs of p(HEMA-co-AGE) cryogels.

To further confirm that a supermacroporous cryogel matrix is produced by the simultaneous polymerization and crosslinking of the monomers HEMA and AGE in cryogenic conditions in the presence of APS/TEMED as an initiator/activator pair, the scanning electron images of the p(HEMA-co-AGE) cryogel were taken and are shown in Figure 3. As can be seen, the p(HEMA-co-AGE) cryogel has some non-porous and thin polymer walls that constitute continuous interconnected pores, providing channels that can be utilized for catalase immobilization. The convective flow of the solution through the pores renders practically negligible resistance to the mass transfer. The specific surface area of the p(HEMA-co-AGE) cryogel was determined to be 2.92 m^2^/g. The prepared p(HEMA-co-AGE) cryogel was opaque, sponge-like, and so elastic that this cryogel could be easily compressed by hand to remove water which had accumulated inside the pores. As the compressed piece of cryogel was submerged in water, it imbibed water within 1–2 s, restoring its original size and shape. The equilibrium swelling degree of the p(HEMA) cryogel was determined to be about 250%. Compared with the p(HEMA) cryogel, the equilibrium swelling degree of the p(HEMA-co-AGE) cryogel decreased to 106%. This is due to the relatively higher hydrophobicity of AGE with respect to HEMA. As can be seen from the chemical structure, the AGE co-monomer was hydrophobic. As it forms a copolymeric cryogel with the HEMA monomer, p(HEMA-co-AGE), the swelling capacity was expected to be lower than the p(HEMA) cryogel, as it was relatively hydrophobic in comparison to the poly(HEMA) cryogels. Therefore, it is anticipated that the swelling degree of the p(HEMA) cryogels will be higher than that of the p(HEMA-co-AGE) cryogels. This result is compatible with similar studies in the literature. For example, Tuzmen et al. reported that the swelling ratio of the p(AAm-AGE)–CB–Fe^3+^ cryogel was 21.6 ± 1.9 g H_2_O/g cryogel [27].

The specific surface area of the p(HEMA-co-AGE) cryogel structures was measured to be 2.92 m^2^/g. Due to the interconnected nature of the macropores’ network of this developed cryogel system, the main advantage was the easy diffusion of large molecules such as enzyme molecules throughout the solid support materials which were to be immobilized, as well as the ready exchange of solute molecules in-and-out of the network where the industrial manufacturing of versatile products is concerned. As we mentioned above, the specific surface area of the p(HEMA-co-AGE) cryogel structures was measured to be 2.92 m^2^/g, which is relatively low in comparison to the poly(HEMA-GMA) cryogel that was reported, by Soomro et al. (2015), to have a specific surface area of 9.2 m^2^/g [28]. The specific surface area of the p(HEMA-GMA) membrane prepared by Arıca and Bayramoğlu (2009) was reported to be 1.43 m^2^/g [29]. Therefore, the surface area of the synthesized p(HEMA-co-AGE) cryogel structures is within rage of the values reported in the literature [28,29].

**Figure 4:**
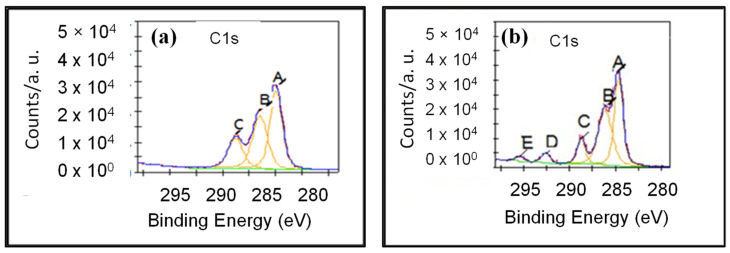
(**a**) C(1s) XPS Spectra of p(HEMA) and (**b**) p(HEMA-co-AGE) cryogels.

The interactions in the covalent and non-covalent attachment of the catalase enzyme with the p(HEMA-co-AGE) and p(HEMA) cryogels were investigated by XPS analysis, and the corresponding C(1s) XPS spectra are shown in Figure 4a,b, respectively. As can be seen, covalent bonding occurs when the epoxy ring in the structure of the p(HEMA-co-AGE) cryogel is opened, upon the catalase enzyme’s immobilization through its amine groups.

According to XPS analysis, in the C(1s) spectrum of the p(HEMA-co-AGE) cryogel, the C-C or C-H bond energies were 284.75 eV, C-O or C-N bond energies were 287.0 eV, and the C=O bond energies of the carbonyl groups were 288.5 eV. The bond energy formed by the opening of the epoxy group via the reaction of the amine groups of the histidine amino acids in the catalase enzyme was 290.5 eV, and the bond energy containing the carboxyl group (-COOH) of the catalase enzyme added to the structure was 295.0 eV [30]. The bond energies of the functional groups from the XPS analysis of the p(HEMA) and p(HEMA-co-AGE) cryogels are summarized in Table 1.

At the same time, the N(1s) XPS spectra were taken to confirm catalase immobilization via the amine–epoxide reaction, and their binding energies of the amine group were determined; the results are presented in Table 2.

In non-specific interactions, the nitrogen atom is neutral (399.0–399.3 eV) and the shift of the energy with covalent bonding (400.6–401.9 eV) indicates that the catalase enzyme reacted with the epoxy group in the p(HEMA-co-AGE) cryogel [31].

### 2.2. Catalase Binding Studies into p(HEMA-co-ADE) Cryogel from Aqueous Solutions

#### 2.2.1. Effect of Flow Rate

**Figure 5:**
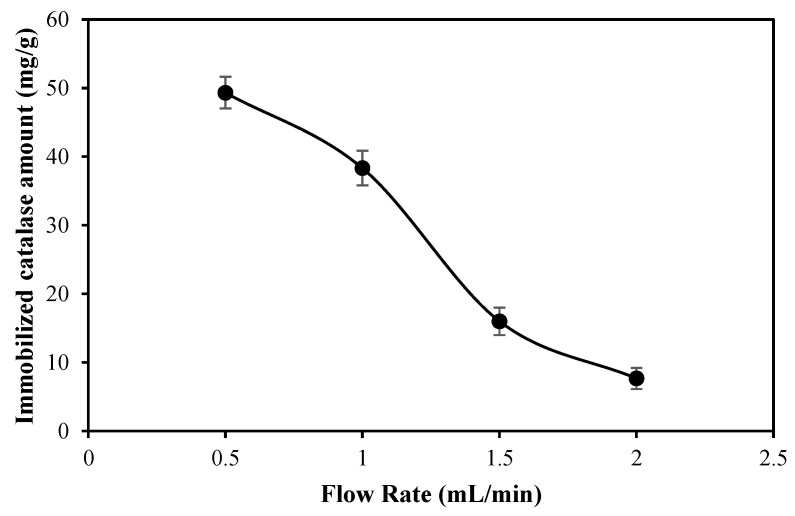
Effect of flow rate on catalase binding capacity of p(HEMA-co-AGE) cryogel [catalase concentration: 1.0 mg/mL in 50 mM pH 6.0 phosphate buffer; T: 25 °C].

To determine the effect of catalase binding onto the p(HEMA-co-AGE) cryogels at different flow rates in the cryogel-containing column is demonstrated in Figure 5. The results showed that as the flow rate increased from 0.5 mL/min to 2.0 mL/min, the amount of catalase binding onto the p(HEMA-co-AGE) cryogel decreased significantly. The reduction in the amount of catalase binding onto the p(HEMA-co-AGE) cryogel could be due to a reduction in the contact time between the catalase molecules and the p(HEMA-co-AGE) cryogel at high flow rates. These results are in agreement with the similar studies reported in the literature [19,32].

As the contact time between the catalase and the cryogel column was extended, the diffusion of the catalase molecules into the pore walls of the cryogel had sufficient time to react with the epoxide groups of the p(HEMA-co-AGE) cryogels because of the presence of the allyl glcydyl ether group. Thus, a higher immobilization amount of catalase onto the p(HEMA-co-AGE) cryogel could be attained at a 0.5 and 1 mL/min flow rate with about 49.3 ± 2.3 and 38.3 ± 2.5 mg/g cryogel, respectively, versus at a 1.5 and 2 mL/min flow rate with about 16.0 ± 2.0 and 7.6 ± 1.5 mg/g cryogel, respectively.

#### 2.2.2. Effects of pH and Initial Catalase Concentration

**Figure 6:**
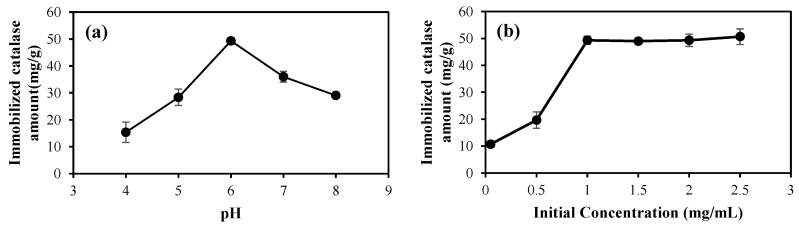
(**a**) Effect of pH on catalase binding amount onto p(HEMA-co-AGE) cryogel [catalase concentration: 1.0 mg/mL; flow rate: 1.0 mL/min; T: 25 °C], and (**b**) effect of initial catalase concentration on catalase binding amount onto p(HEMA-co-AGE) cryogel [reaction conditions: 50 mM phosphate buffer pH 6.0; flow rate: 1.0 mL/min; T: 25 °C].

To determine the effects of pH and the initial catalase concentration onto the p(HEMA-co-AGE) cryogels, the immobilization reactions were carried out at different pH solutions and catalase concentrations, and the corresponding graphs are illustrated in Figure 6a,b, respectively. The used buffer solutions were acetate buffers of pH 4.0–5.0, and phosphate buffers of pH 6.0–8.0. The covalent binding between the epoxy group of the p(HEMA-co-AGE) cryogel with catalase occurred as a result of the reaction of the amine groups of the histidine in the catalase structure, therefore, at the low pH medium, the amine groups were protonated and lost their nucleophilic properties. As can be seen in Figure 6a, the highest loading amounts of catalase attained was 49.3 ± 1.2 mg/g at pH 6.0. At higher solution media, the activity of the amine groups was also reduced due to the basic nature of the medium as relatively more hydroxy groups could compete and reduce the nucleophilic activity of the amine groups of the enzyme. Thus, the amount of enzyme immobilization at pH 7 and 8 was less than that of pH 6. As the catalase isoelectric point should be about 5, this is reasonable. In addition to this, the amino acid side chains of the catalase molecules can also cause secondary interactions (such as hydrogen bonding, ionic interactions, hydrophobic interactions) with the related functional groups in the cryogel structure. It was also found that catalase adsorption on the p(HEMA) cryogel was negligible at all pH ranges studied as no reactive binding groups on the p(HEMA) cryogel were available to interact with the catalase molecules. Therefore, non-specific binding may result from the diffusion of catalase molecules into the swollen cryogel and weak interactions such as the van der Waals interaction, as well as the hydrogen bonding between the catalase and hydroxyl groups on the surface of the p(HEMA) cryogel which may exist, however, this non-chemically bounded catalase can be readily removed during washing and through small pH changes.

Figure 6b shows the effect of the initial concentration of catalase on the catalase binding amount onto the p(HEMA-co-AGE) cryogel. The adsorption values increased with the increasing concentration of catalase, and a saturation value was achieved at a catalase concentration of 1.0 mg/mL, which represents the saturation of the active binding cavities on the p(HEMA-co-AGE) cryogel. A maximum immobilization amounts of 49.3 ± 1.5 mg/g cryogel was attained for all the initial catalase concentrations ≥1 mg/mL.

### 2.3. Kinetic Parameters

Kinetic parameters such as the Michaelis constant (K_m_) and V_max_ for the free and immobilized catalase on the p(HEMA-co-AGE) cryogel were determined by Lineweaver–Burk plots using H_2_O_2_ as substrate. V_max_ defines the maximum rate at which the enzyme is saturated with its substrate, so this parameter reflects the properties of the binding enzyme, although it is affected by diffusion restrictions. K_m_ is defined as the substrate concentration at a reaction rate of 1/2 Vmax. Km is an indicator of the affinity of the enzyme to its substrate. Here, it was found that Km and Vmax values were significantly influenced by the immobilization of catalase on the p(HEMA-co-AGE) cryogel, as expected. The Km value of 46 mM for the free catalase and 19 mM for the immobilized catalase was calculated. So, there was an approximate 2.5-fold decrease in the K_m_ value for immobilized catalase onto the cryogel. In addition, the V_max_ value of the free catalase, 6.6 × 10^4^ U/mg enzyme, was found to be higher than the immobilized catalase, 1.74 × 10^3^ U/mg enzyme. As expected, the K_m_ and V_max_ values were significantly changed by the immobilization of catalase onto the p(HEMA-co-AGE) cryogel. This change in the enzymes’ affinity for its substrate is likely due to structural changes in the enzyme or the lower accessibility of the substrate to the active site of the immobilized enzyme. It may also be inferred that the enzyme structure may have been changing within the p(HEMA-co-AGE) cryogel matrix upon immobilization. Therefore, the change in affinity could be attributed to the structural change of the enzyme which can reduce the accessibility of the substrate to the active site [33]. The V_max_ value of the immobilized catalase was lower than that of the free catalase due to the loss of activity. This may be as a result of the interactions of the enzyme functional groups attached to the cryogels, or because the contact regions of the enzyme and the cryogel may lead to deformations of the enzymatic conformations or arrangements. Similar to these results, the reduction in the K_m_ and V_max_ values of the enzyme after immobilization with respect to free enzymes has been reported in the literature [34,35,36]. For instance, Inanan reported that the Km value was decreased 2.4-fold when compared with the free enzymes value [37]. Çetinus et al. revealed that the immobilized enzyme on chitosan beads also exhibited a decreased value, approximately by 2.0 times in the K_m_ value [38]. Similarly, Erol et al. reported that the K_m_ value was decreased by approximately 2.0 times after immobilization on the poly(HEMA-GMA) cryogels [39]. In addition, the V_max_ value for the immobilized enzyme was about 1.8-fold higher than that of the free counterpart. Generally, the enhancements in the V_max_ values corroborated with the substrate diffusion ratio, conformation changes, and cooperative effects of the enzymes [40].

### 2.4. Effect of Temperature and pH on the Catalytic Activity

**Figure 7:**
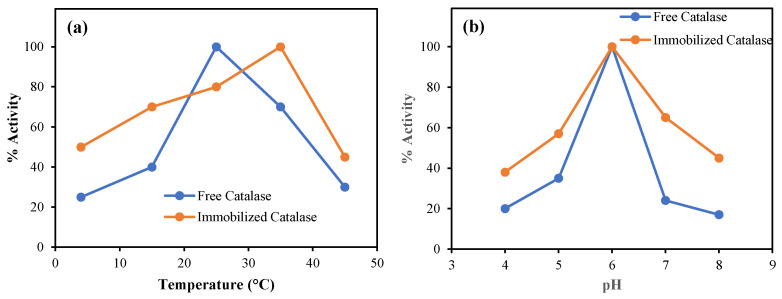
(**a**) Effects of temperature on % activity of free catalase and immobilized catalase enzymes onto p(HEMA-co-AGE) cryogels [reaction conditions: 50 mM phosphate buffer, pH 6.0], and (**b**) effects of pH on % activity of free and immobilized catalase enzymes onto p(HEMA-co-AGE) cryogels [reaction conditions: 50 mM phosphate buffer, T = 35 °C].

The temperature dependence of the activities of free and immobilized catalase were studied in 50 mM phosphate buffer (pH 6.0) in a temperature range of 4–45 °C. The temperature profiles of the free and immobilized catalase are shown in Figure 7a. The change in the % activity, as expected, increased with the temperature up to an optimum reaction temperature of 25 °C, and decreased almost linearly at temperatures ≥ 25 °C for the free enzyme. Interestingly, the % activity of the immobilized enzyme increased almost linearly up to 35 °C, and then reduced at temperature higher than 35 °C. Consequently, it is apparent that the p(HEMA-co-AGE) cryogel network provides a sheltered environment for the immobilized enzyme to afford higher enzymatic activity at a higher temperature with respect to the free enzyme. Overall, it can be said that the catalase activity of the immobilized enzyme onto the p(HEMA-co-AGE) cryogel was higher than that of the free catalase for each studied temperature, and the optimum temperature was found to be at about 25 °C for free catalase and 35 °C for the immobilized catalase enzymes.

The effect of pH on the % activity of free and immobilized catalase for H_2_O_2_ degradation was studied at various pHs at 35°C. The reactions, as mentioned earlier, were carried out in acetate (pH 4 and 5) and phosphate buffers (pH 6–8), and the results are presented in Figure 7b. As can be seen, the optimum pH for the free and immobilized enzymes was the same, at pH 6.0. Again, the effect of pH on the immobilized catalase was less than the free enzyme as, in all the studied pH solutions, the % activity of the immobilized catalase enzyme was higher than the free enzymes except at pH 6, where the activity of both enzymes was the same. Yet again, the p(HEMA-co-AGE) enzyme provides protection for the immobilized enzyme against relatively harsh pH conditions, e.g., at pH 4, 5, 7, and 8, keeping in mind that the optimum pH for the catalase enzyme is 6.

### 2.5. Reusability and Stability of Catalase-Immobilized p(HEMA-co-AGE) Cryogel

**Figure 8:**
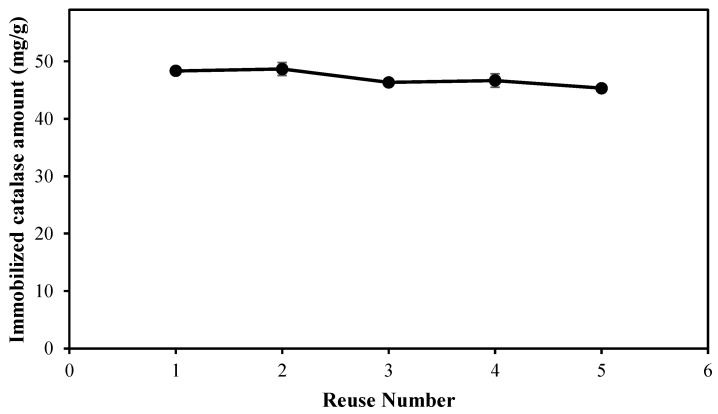
Reusability of p(HEMA-co-AGE) cryogel [flow rate: 1.0 mL/min; catalase concentration: 1.0 mg/mL; T: 25 °C].

In order to show the stability and reusability of the catalase-immobilized p(HEMA-co-AGE) cryogel, the adsorption–desorption cycle was repeated five times using the same cryogel. For sterilization after one adsorption–desorption cycle, the catalase-immobilized cryogel was washed with 50 mM NaOH solution for 30 min, washed with DI water for 30 min, and then equilibrated with the phosphate buffer at pH 7.0 for the subsequent adsorption–desorption cycle. After performing the enzymatic assay reaction, as stated in Section 4.4, to determine the stability, the cryogel was very stable and maintained its adsorption capacity as the recovery ratio of catalase was calculated as 93.8 ± 1.2% after 5th use as illustrated in Figure 8.

Therefore, it is obvious that the enzyme-immobilized p(HEMA-co-AGE) cryogel can be used many times without significantly decreasing its catalase-binding capacities significantly.

## 3. Conclusions

Cryogels are polymeric networks that are synthesized under cryogenic gelation conditions for different biomedical applications due to their interesting porous structure and the ease of their preparation. The physical and chemical properties of cryogels can be tuned in advance by appropriately choosing starting polymers and monomers to bestow them with many advantages. As cryogels are interconnected supermacroporous structures with high mechanical and chemical stability, they can be readily used in catalytic reactions, tissue engineering, separation, and filtration purposes. Therefore, here we demonstrated that the catalase enzyme can be readily immobilized onto a newly synthesized p(HEMA-co-AGE) cryogel. It was also demonstrated that the p(HEMA-co-AGE) cryogel has epoxide in the structures to be used or modified for further applications, e.g., to link some biomolecules. The developed cryogel structures were shown to be effective in the immobilization of the catalase enzyme which performed better than the free enzymes at different pHs and temperatures.

In the literature, Fe^3+^-immobilized poly(acrylamide-allylglycidyl ether) [p(AAm-AGE)]-based cryogels carrying Cibacron Blue F3GA, employed as a novel class of adsorbents for the metal-chelated affinity immobilization of catalase, were reported [27]. By the radical polymerization of acrylamide and allylglycidyl ether, the p(AAm-AGE) cryogels were prepared and Cibacron Blue F3GA was covalently attached to the p(AAm-AGE) cryogels as a chelating agent. Then, Fe^3+^ ions were immobilized onto the cryogel support matrix via coordinate bonding with this chelating agent. Maximum adsorption capacities were reported as being 75.7 ± 1.2 mg/g for the p(AAm-AGE)–CB–Fe^3+^ cryogels and 60.6 ± 1.0 mg/g for the p(AAm-AGE)–CB cryogels, respectively. Here, 48 mg/g catalase onto the p(HEMA-co-AGE) cryogels were immobilized without any additional impurities such as Cibacron Blue F3GA and Fe^3+^ ions. The monolith cryogels most used as a stationary phase in affinity chromatography were prepared using copolymers of poly-acrylamide (PAAm), glycidyl methacrylate (GMA), and ethylene glycol dimethacrylate (EDMA) [41]. The PAAm-Alg-AGE was synthesized by the copolymerization of AGE, acrylamide, and alginate with N, N’-Methylenebisacrylamide, and Ca(II) as the crosslinking agents, and the AGE has a conjugated epoxy group that provided the immobilization of P-Tyr.

The advantages, such as facile diffusion and easy mass transfer due to their macroporous structure, of the p(HEMA-co-AGE) cryogels also make them promising candidates for other biotechnological applications including the isolation, immobilization, and purification of biomolecules, separation of cells, drug delivery systems, and so on.

## 4. Materials and Methods

### 4.1. Materials

Commercial catalase enzyme (EC. 1.11.1.6), hydrogen peroxide, ethylene glycoldimethacrylate (EGDMA, 98%), 2-hydroxyethyl methacrylate (HEMA, 98%), allyl glycidyl ether (AGE, ≥99%), N,N′-methylenebisacrylamide (MBAAm, 99%), N,N,N′,N′-tetramethylene diamine (TEMED, ≥99.5%)), ammonium persulfate (APS, ≥99.99%), polyvinyl alcohol (PVA), isopropyl alcohol (Bioreagent, ≥99.5%), ethanol (99.8%), sodium acetate (>99%), sodium chloride (NaCl, ACS reagent, ≥99.0%), potassium dihydrogen phosphate, potassium hydrogen phosphate, and hydrogen peroxide solution (H_2_O_2_, 30%) were obtained from Sigma (Steinheim, Germany).

### 4.2. Synthesis and Characterization of p(HEMA-co-AGE) Cryogels

The synthesis of cryogels was carried out by free radical polymerization under cryogenic conditions. To prepare p(HEMA-co-AGE) monolithic cryogel, 0.283 g MBAAm was dissolved in 10 mL distilled water, and 1.5 mL HEMA and 100 µL AGE mixture was dissolved in 3.5 mL distilled water. These reagents, chilled in the ice bath in separate beakers, were then mixed and 20 mg of APS was added. Next, 25 µL of TEMED was added to the mixture. Then, 3.5 mL of this cryogel precursor was placed into the syringe (5.0 mL volume, 0.8 cm diameter) so that the tip of the syringe was sealed with parafilm and kept at −12 °C for 24 h. After cryogellation, the p(HEMA-co-AGE) cryogels-containing syringe was allowed to thaw at room temperature, was then washed with 200 mL of distilled water in a pH 7.0 phosphate buffer solution containing 0.02% sodium azide (0.1 M), and stored at 4 °C.

The chemical structure of the p(HEMA-co-AGE) cryogel was corroborated by FTIR-ATR (Fourier Transform Infrared-Attenuated Total Reflection) Spectrophotometer analysis. The cryogel samples (~0.1 g) were dried and placed in the device in solid form, and the FTIR-ATR spectra were taken.

The surface morphology of the cryogels was examined using scanning electron microscopy (SEM, Phillips XL-30S FEG, Almelo, The Netherlands).

Surface area measurements of the synthesized cryogels were done using the Brunauer–Emmett–Teller method using a surface area and porosity analyzer (BET, Micromeritics Gemini V). For this purpose, measurements were taken after drying the cryogel in an oven at 60 °C and grinding them into powder.

X-ray Photoelectron Spectrophotometer (XPS, Thermo Scientific K-Alpha) device was used to determine the binding nature of catalase enzyme onto the synthesized cryogels. The XPS analyses were done on the powdered dried samples.

To determine the swelling ratio of p(HEMA-AGE) cryogels, the cryogels were dried at 60 °C for 24 h. Then, the dried cryogels were weighed and kept in distilled water at 25 °C, the amount of adsorbed water was measured at certain time intervals by weight increase measurements, and the swelling ability of the cryogels were determined. Swelling ratio% of the cryogels were calculated using Equation (1).

Swelling ratio% = (m_wet cryogel_ − m_dry_
_cryogel_)/m_dry_ _cryogel_ (1)

where, m_wet cryogel_ and m_dry cryogel_ are the weights of the cryogel in swollen and dry states, respectively.

### 4.3. Catalase Immobilization onto p(HEMA-co-AGE) Cryogels and Desorption of Catalase from p(HEMA-co-AGE) Cryogels

The binding of catalase onto p(HEMA-co-AGE) cryogel was investigated by frontal adsorption experiments. The p(HEMA-co-AGE) cryogel was washed with 30 mL of DIwater and then equilibrated with 50 mM phosphate buffer (pH 7.0). The catalase solution was then pumped using a peristaltic pump (WATSON MARLOW sci 400) into the cryogel column (total cryogel mass: 370 mg). The binding of catalase onto the cryogel column was followed by measuring the decrease in the absorbance values in catalase concentration using a UV-Visible spectrophotometer (Thermo Scientific, Waltham, MA, USA) at 280 nm. The effects of flow rate, catalase concentration, and pH on the binding capacity were investigated. To determine the effect of flow rate, the flow rate was varied in the range of 0.5–2.0 mL/min. To determine the effect of pH on catalase binding, the solution pH was varied in pH 4.0–8.0 range. To assess the effect of catalase’s initial concentration on binding, the catalase’s initial concentration was changed in 0.1–2.0 mg/mL range.

In studies of catalase binding to synthesized cryogels, initial concentration and medium pH parameters were investigated and maximum catalase binding conditions of cryogels were optimized. In order to investigate the effect of pH on catalase immobilization on polymeric cryogels, catalase solutions were prepared at a concentration of 0.5 mg/mL at different pHs (pH 4.0–5.0 acetate buffer; pH 6.0–8.0 phosphate buffer; 0.1 M). Sample of 1.0 mL of these solutions was taken, and their absorbance was read at 280 nm. Then, the enzyme solution was passed through the cryogel column for 18 h with the help of a peristaltic pump. At the end of this time period, the absorbances value of the solutions passed through the column were read at 280 nm. The amount of catalase bound to the cryogel versus the pH values was plotted and the optimum pH value of the enzyme catalase bound to the cryogel structures was determined. Experiments were performed in triplicate. Equation (2) was used to determine the amount of binding in the covalent immobilization of catalase enzyme to cryogels;

Q = [(C_o_ − C) V]/m (2)

where Q is the amount of catalase adsorbed onto unit mass of cryogels (mg/g), C_o_ and C are the concentration of catalase in the initial solution and in the aqueous phase after treatment for certain period, respectively (mg/mL), V is the volume of the aqueous phase (mL), and m is the mass of the cryogels used (g).

To determine the effect of catalase’s initial concentration on the immobilization efficiency onto cryogels, the experiments were performed at 25 °C in 5 mL volume, at pH 7.0 in PBS at catalase concentrations of 0.1, 0.25, 0.5, 0.75, 1.0, and 2.0 mg/mL. As stated above, the amounts of catalase bound onto the cryogel structures were calculated for each enzyme concentration. The amounts of catalase bound onto the cryogel versus the concentration values were plotted and the binding amount of the catalase onto the cryogel structures was determined.

In order to determine the reusability of p(HEMA-co-AGE) cryogels, the catalase adsorption and desorption cycle was repeated five times. The catalase desorption from p(HEMA-co-AGE) cryogels was carried out with 1.0 M NaSCN solution at pH 8.0. In a typical desorption experiment, 30 mL of the desorption agent was pumped through the cryogel column at a flow rate of 1.0 mL/min for 2 h. The final catalase concentration in the desorption medium was determined spectrophotometrically at the measurement of the absorbance values at 280 nm. The desorption ratio was calculated from the amount of catalase adsorbed on the cryogel and the final catalase concentration in the desorption medium. In order to test the reusability of p(HEMA-co-AGE) cryogels, the catalase adsorption–desorption procedure was repeated five times using the same cryogel column. In order to regenerate and sterilize, the column was washed with 50 mM NaOH solution after each adsorption–desorption cycle.

### 4.4. Activity Assays of Free and Immobilized Catalase Enzymes

Catalase activity was also determined spectrophotometrically by direct measurement of the decrease in the absorbance of H_2_O_2_ at 240 nm, due to its decomposition by the enzyme. H_2_O_2_ solutions in 5–30 mM range were used to determine the activities of both free and immobilized enzymes. A 4-mL portion of the reaction mixture was preincubated at 25 °C for 10 min, and the reaction was started by adding 50 µL of catalase solution (100 µg/mL). The decrease in absorbance at 240 nm was recorded after 5 min. The rate of change in the absorbance was calculated from the initial linear portion using a calibration curve that was constructed from absorbance values of H_2_O_2_ solutions at various concentrations (5–30 mM) at 240 nm. One unit of activity is defined as the decomposition of 1 µmol/min of H_2_O_2_ at 25 °C and pH 7.0. These activity assays were carried out over the pH range 4.0–8.0, and the temperature range of 4–65 °C to determine the effect of pH and temperature on stability of the free and immobilized enzymes. The effect of substrate concentration was tested in the 5–30 mM H_2_O_2_ range. The effects of pH and temperature are presented in a normalized form, with the highest value of each set being assigned the value of 100% activity. The thermal stability of free and immobilized catalase of cryogel were performed at 55 °C in acetate buffer (0.1 M, pH 5.0). For storage stability of free and immobilized catalase enzymes, the materials were stored in 0.1 M of acetate buffer (pH 5.0) at 4 °C for 45 days.

## Figures and Tables

**Table 1:** C(1s) XPS analysis.

Scanning Area	Bond Type	Energy (eV)
A	C-C, C-H	284.75
B	C-O or C-N	287
C	C=O	288.5
D	C-N	290.5
E	COOH	295

**Table 2:** N(1S) XPS spectra analysis of p(HEMA) and p(HEMA-co-AGE).

p(HEMA)	p(HEMA-co-AGE)
399.08	402.64

## Data Availability

The data presented in this study are available on request from the corresponding authors.
